# *GhCLCg-1*, a Vacuolar Chloride Channel, Contributes to Salt Tolerance by Regulating Ion Accumulation in Upland Cotton

**DOI:** 10.3389/fpls.2021.765173

**Published:** 2021-10-15

**Authors:** Wei Liu, Junping Feng, Wenyu Ma, Yang Zhou, Zongbin Ma

**Affiliations:** ^1^Collaborative Innovation Center of Henan Grain Crops, College of Agronomy, Henan Agricultural University, Zhengzhou, China; ^2^Weinan Vocational and Technical College, Weinan, China; ^3^Hainan Key Laboratory for Biotechnology of Salt Tolerant Crops, College of Horticulture, Hainan University, Haikou, China

**Keywords:** chloride channel, *Gossypium hirsutum*, vacuolar, ion content, salt tolerance

## Abstract

Soil and freshwater salinization is increasingly becoming a problem worldwide and has adversely affected plant growth. However, most of the related studies have focused on sodium ion (Na^+^) stress, with relatively little research on chloride ion (Cl^–^) stress. Here, we found that upland cotton (*Gossypium hirsutum*) plants accumulated Cl^–^ and exhibited strong growth inhibition under NaCl or KCl treatment. Then, a chloride channel gene (*GhCLCg-1*) was cloned from upland cotton. Phylogenetic and sequence analyses indicated that *GhCLCg-1* was highly homologous to *AtCLCg* and also have conserved voltage_CLC and CBS domains. The subcellular localization assay showed that GhCLCg-1 was localized on the vacuolar membrane. Gene expression analyses revealed that the expression of *GhCLCg-1* increased rapidly in cotton in response to chloride stress (NaCl or KCl), and the transcript levels increased as the chloride stress intensified. The overexpression of *GhCLCg-1* in *Arabidopsis thaliana* changed the uptake of ions with a decrease of the Na^+^/K^+^ ratios in the roots, stems, and leaves, and enhanced salt tolerance. In contrast, silencing *GhCLCg-1* in cotton plants increased the Cl^–^ contents in the roots, stems, and leaves and the Na^+^/K^+^ ratios in the stems and leaves, resulting in compromised salt tolerance. These results provide important insights into the toxicity of chloride to plants and also indicate that *GhCLCg-1* can positively regulates salt tolerance by adjusting ion accumulation in upland cotton.

## Introduction

Salinization is a serious environmental problem worldwide. During the last few decades, many fresh water regions have been affected by increasing salinity (Kaushal, 2016). The causes of salinization include alterations to freshwater flows, irrigation, wastewater treatment, sea level increases, and the use of salt for deicing roads (Herbert et al., 2015). The application of deicing salts, such as NaCl, CaCl_2_, and MgCl_2_, has increased the salinization of water and soil, which has directly and negatively affected natural environments (Buss et al., 2020). Because chloride salts are commonly used, the chloride ion (Cl^–^) may be useful as a tracer for deicing salts (Thunqvist, 2004). Recent studies proved that the widespread use of road salt has resulted in increased chloride concentrations in lakes and rivers (Thunqvist, 2004; Gutchess et al., 2016; Laceby et al., 2019). Vehicular traffic may be responsible for transferring salt into the adjacent roadside, where the salt may seep into the soil and the underlying water (Fay and Shi, 2012).

Salinization is extremely detrimental to sustainable agricultural production worldwide (Saghir et al., 2002). Sodium chloride (NaCl), which is the most ubiquitous salt, induces cellular osmotic and ionic stresses (Deinlein et al., 2014; Nie et al., 2015). To date, studies on the physiological and molecular mechanisms underlying plant salt tolerance have mostly focused on Na^+^ toxicity and adaptations, but there has been relatively little research on salt damage caused by Cl^–^ (Teakle and Tyerman, 2010; Li et al., 2016). The previous studies have confirmed that sodium/proton (Na^+^/H^+^) antiporters (NHXs) are involved in the regulation and stabilization of Na^+^ under salt stress in plants and improved the salt tolerance of plants (Ma et al., 2020; Feng et al., 2021).

The chloride ion is one of the essential trace elements in plants. More specifically, it enhances plant growth and development (Broyer et al., 1954; Wei et al., 2019), while also affecting stomatal movement, cell osmotic pressure, electrical charge balance, cell turgor pressure, and intracellular pH (Franco Navarro et al., 2015). Despite its beneficial effects on plants, under salt stress conditions, Cl^–^ is a major toxic element in the cytosol of plant cells, where it accumulates excessively, especially in shoots, thereby adversely affecting growth (Munns and Tester, 2008; Weg et al., 2017). Plants counter these stresses *via* the elimination of excess ions across the plasma membrane or the intracellular vacuolar compartmentalization to decrease the effective Cl^–^ levels inside cells, particularly in the aerial tissues (Zhang et al., 2011). Earlier research revealed that the vacuolar chloride channel CLCg selectively transports chloride across the vacuolar membrane to enhance the tolerance to chloride salts (Nguyen et al., 2015). A recent study indicated *GhCLC4/15* expression is up-regulated in the roots and leaves of cotton seedlings under salt stress (Liu et al., 2020). Additionally, the vacuolar chloride channel CLCc maintains chloride homeostasis. The *Arabidopsis thaliana* mutant *atclc-c* reportedly exhibits increased sensitivity to NaCl (Jossier et al., 2010). In tobacco (*Nicotiana tabacum*), the Cl^–^ content is obviously lower in *NtCLC2*-silenced plants than in control plants (Zhang et al., 2018). The overexpression of the vacuolar chloride channel gene *GmCLC1* in soybean can increase the segregation of Cl^–^ in the roots and decrease the transport of Cl^–^ to the shoots (Wei et al., 2016). These findings suggest that CLCs can take up Cl^–^ to increase salt tolerance at high salt concentrations, and the resulting accumulation of Cl^–^ in plants may minimize the effects of chloride on the environment.

Upland cotton (*Gossypium hirsutum*) is a widely grown fiber crop and has been used as a pioneer crop in regions with saline and alkaline land (Wang N. et al., 2016; Wang X.G. et al., 2016). In this study, the potential effects of chloride concentrations on cotton plants were analyzed. We treated cotton plants with different chloride salts (NaCl or KCl) and observed the resulting damage. And we also found the vacuolar chloride channel *GhCLCg-1* was activated in plants exposed to chloride salt concentrations by quantitative real-time polymerase chain reaction (PCR) (qRT-PCR). Furthermore, overexpression in *A. thaliana* and virus-induced gene silencing (VIGS) technology in cotton were used to characterize the function of *GhCLCg-1* in cotton response to chloride salt stress. The objectives of this study were to determine the toxicity of chloride in cotton plants and prove the important role of *GhCLCg-1* in cotton resistance to salinity.

## Materials and Methods

### Plant Materials and Growth Conditions

*Gossypium hirsutum* L. acc. TM-1 seeds were soaked in sterile water to promote germination. After a 16-h incubation at 30°C, the germinating seeds were transferred to vermiculite-filled pots in a greenhouse (16-h light/8-h dark photoperiod at 23°C). When the cotyledon expanded, uniformly growing seedlings were selected, rinsed clean with running water, and transferred to a hydroponic tank containing Hoagland solution, which was refreshed weekly. To induce salinity stress, the cotton seedlings were grown to the three-leaf stage and then treated with Hoagland solution containing 50, 100, 150, or 200 mM NaCl or 200 mM KCl (treatment group). The seedlings in the control group were treated with Hoagland solution lacking NaCl or KCl. Root, stem, and leaf samples were collected at 0, 1, 3, and 6 h after treatments. The samples were rinsed with distilled water 3–5 times and then immediately frozen in liquid nitrogen and stored at −80°C for the subsequent total RNA extraction. Each treatment was completed with three biological replicates.

The wild-type (WT) *A. thaliana* plants used in this study were Columbia (Col-0). After a 2-day vernalization at 4°C, the *A. thaliana* seeds were surface sterilized by soaking in 75% ethanol for 3–5 min. After they were washed with sterile distilled water six times, the seeds were placed in plates containing Murashige and Skoog (MS) medium supplemented with 3% sucrose and 0.7% agar (pH adjusted to 5.8). The plates were incubated at 21°C with a 16-h light/8-h dark photoperiod. After 2 weeks, the *A. thaliana* seedlings were transferred to pots filled with vermiculite and nutritive soil (1:1, v/v) and placed in a greenhouse (16-h light/8-h dark photoperiod at 21°C).

### Gene Cloning and Sequencing

Total RNA was isolated from TM-1 seedlings using the RNAprep Pure Polysaccharide Polyphenol Plant Total RNA Extraction Kit (Tiangen, Beijing, China). The RNA concentration was determined using the NanoDrop 2000 microvolume spectrophotometer (Thermo Fisher Scientific, Waltham, MA, United States), whereas RNA integrity was assessed by 1% agarose gel electrophoresis. First-strand cDNA was synthesized from 1 μg RNA using the PrimeScript^TM^ II first Strand cDNA Synthesis Kit (TaKaRa, Dalian, China). To clone the vacuolar chloride channel gene in upland cotton, the BlastP and tBlastN programs were performed against the *G. hirsutum* genome database^1^ using the *A. thaliana* CLC*g* amino acid sequence as the query. The coding sequences of gene IDs GH_A06G0574 and GH_D06G0541 as the best match were retrieved and gene-specific primers were designed. Full-length cDNAs were amplified using the GhCLCg-1F/R primer pair (Supplementary Table 1) and the KOD-Plus-Neo DNA polymerase (TaKaRa, Dalian, China). The PCR products were purified using the FastPure Gel DNA Extraction Mini Kit (Vazemy Biotech Co., Ltd.) and then cloned using the pEASY^®^-Blunt Cloning Kit for the subsequent transformation of *Trans1*-T1 *Escherichia coli* competent cells (TransGen Biotech). Positive clones cultured at 37°C were analyzed by sequencing to verify they were correctly transformed.

### Phylogenetic Tree Construction and Sequence Analysis

*Arabidopsis thaliana* CLC amino acid sequences were downloaded from TAIR database^2^. Seven AtCLC sequences along with *G. hirsutum* GhCLCg-1A and GhCLCg-1D (Supplementary Table 2) were used to construct a phylogenetic tree according to the neighbor-joining method of the MEGA-X software, with pairwise deletion and 1,000 bootstrap replicates. Conserved domains were detected using InterPro^3^ and gene structures were examined using TBtools (Chen et al., 2020). Amino acid sequences were aligned using the DNAMAN program.

### Quantitative Real-Time Polymerase Chain Reaction Analysis

Total RNA was isolated from the roots, stems, and leaves using the RNA Extraction kit (Tiangen). The HiScript^®^ II Q Select RT SuperMix for qPCR (+gDNA wiper) (Vazemy Biotech Co., Ltd.) was used to synthesize cDNA from approximately 1 μg total RNA. Gene-specific primers were designed according to conversed sequences in *GhCLCg-1A* and *GhCLCg-1D* (Supplementary Table 1). The qRT-PCR analysis was performed using the LightCycler 480 system (Roche, Switzerland) and the ChamQ Universal SYBR qPCR Master Mix (Vazemy Biotech Co., Ltd.). The *GhHIS3* gene served as an internal control (Ma et al., 2020). Three biological replicates were analyzed for each sample. Relative gene expression levels were calculated according to the 2^–ΔCT^ method (Livak and Schmittgen, 2001).

### Gene Overexpression Plasmid Construction

The full-length *GhCLCg-1A* and *GhCLCg-1D* open reading frames without a stop codon were amplified by PCR using primers with *Bam*HI and *Sma*I sites (Supplementary Table 1) and then inserted into the *Bam*HI/*Sma*I-digested pCAMBIA2300-35S-GFP-HA plasmid, which contains the CaMV 35S promoter. The resulting recombinant plasmids were inserted into *Trans1*-T1 competent cells (TransGen Biotech). The accuracy of the inserted sequences in the transformants was confirmed by sequencing. The recombinant plasmids carrying the GhCLCg-1A-GFP and GhCLCg-1D-GFP constructs were inserted into *Agrobacterium tumefaciens* strain GV3101 cells *via* a heat shock protocol. The *E. coli* and *A. tumefaciens* strains containing the overexpression plasmids were stored at −80°C.

### Subcellular Localization of *GhCLCg-1A/D*

A transient expression system using *A. thaliana* mesophyll protoplasts was prepared as previously described (Sheen, 2001; Feng et al., 2021). Protoplasts were co-transfected with 10 μg GhCLCg-1A-GFP/GhCLCg-1D-GFP recombinant plasmids and 10 μg vacuolar marker protein δ-TIP-RFP (Jauh et al., 1998) according to a polyethylene glycol-mediated transfection method (Yoo et al., 2007). After incubating the protoplasts at room temperature for 12–20 h in darkness, the green fluorescent protein (GFP) and red fluorescent protein (RFP) signals were detected using the FV1200 confocal laser scanning microscope (Olympus, Japan).

### Virus-Induced *GhCLCg-1* Silencing in Upland Cotton

Based on the *GhCLCg-1A* and *GhCLCg-1D* conserved sequences, a 430-bp fragment was amplified by PCR from cotton cDNA using the GhCLCg-1-p-F/R primers (Supplementary Table 1) and inserted between the *Sac*I and *Eco*RI restriction sites in the VIGS vector pTRV2 of the ClonExpress^®^ Ultra One Step Cloning Kit (Vazemy Biotech Co., Ltd.). Plasmids from the positive transformants carrying pTRV1 (helper plasmid) and *TRV:GhCLCg-1* were inserted into *A. tumefaciens* strain GV3101 cells *via* a heat shock protocol. Similarly, we constructed a visual marker (*TRV:CLA*, where CLA is cloroplastos alterados) to monitor the silencing efficiency. Additionally, *TRV:00* (empty vector) was used as the negative control. The VIGS experiments were performed by the tobacco rattle virus (TRV) system as previously described (Pang et al., 2013).

Two fully expanded cotyledons of cotton seedlings were used for the *A. tumefaciens* infiltration. At 10 days after the transformation, the true leaves of *TRV:CLA*-silenced cotton plants exhibited signs of albinism. The second true leaves, stems, and roots were harvested from at least three randomly selected *TRV:00* and *TRV:GhCLCg-1* cotton plants for an RNA isolation. The efficiency of *GhCLCg-1* silencing was evaluated by qRT-PCR, with *GhHIS3* used as the internal control (Ma et al., 2020). The *TRV:00* and *TRV:GhCLCg-1* plants were then treated with 200 mM NaCl for 3-day, the plants were photographed and their roots, stems, and leaves were collected for an analysis of their Cl^–^, Na^+^, and K^+^ contents. The assays were completed with three biological replicates.

### *GhCLCg-1* Overexpression in *Arabidopsis thaliana*

Because *GhCLCg-1A* and *GhCLCg-1D* have highly similar sequences, we used *GhCLCg-1A* to generate transgenic *A. thaliana* plants overexpressing *GhCLCg-1*. The recombinant plasmid carrying the GhCLCg-1A-GFP fusion construct was inserted into *A. thaliana* plants using the floral dip transformation method (Clough and Bent, 1998). Homozygous plants were selected on MS medium containing 50 μg/ml kanamycin. The T_1_ generation plants were examined by PCR to confirm the positive lines were transformed correctly. Homozygous T_3_ generation progenies were analyzed by reverse transcription PCR using the GhCLCg-1-Q-F/R primers (Supplementary Table 1). Seeds from homozygous T_3_ plants were placed on MS agar medium (control group) or MS agar medium supplemented with 100 mM NaCl (salt treatment group). The seedlings were photographed after 3 weeks. Additionally, the taproot length was measured using a vernier caliper. Nine plants per line were examined and their tissues were harvested for an analysis of the Cl^–^, Na^+^, and K^+^ contents.

### Analysis of Ion Contents

The VIGS plants and transgenic *A. thaliana* plants overexpressing *GhCLCg-1* were collected and incubated at 105°C for 10 min to denature enzymes and then at 75°C to achieve a constant weight. The samples were subsequently ground to a powder. To measure the Cl^–^ content, a 50-ml Erlenmeyer flask was washed with 5% HNO_3_. After drying, 0.1 g powder was added to the flask and resuspended with 15 ml boiled deionized water. The solution was cooled to room temperature and then filtered. The filtrate was collected in a 25-ml volumetric flask to a constant volume. The solution was analyzed using an ion chromatography system (ICS-5000) (Thermo Fisher Scientific, Waltham, MA, United States) to measure the Cl^–^ content. The experiment was repeated three times.

To measure the Na^+^ and K^+^ contents, 0.1 g powder was treated with nitric acid (HNO_3_), hydrogen peroxide (H_2_O_2_), and hydrofluoric acid (HF) and then microwaved (i.e., digested) before being analyzed using an inductively coupled plasma optical emission spectrometry system (ICAP-7400) (Thermo Fisher Scientific, Waltham, MA, United States). Three biological replicates were analyzed for each sample.

## Results

### Uptake and Effect of Chloride in Upland Cotton Plants

**FIGURE 1:**
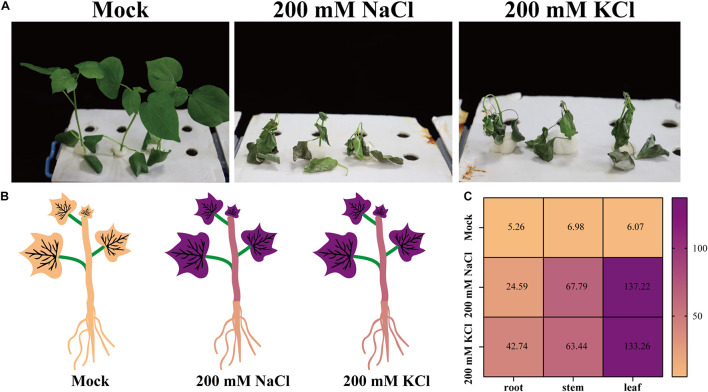
The resulting phenotypes and chloride uptake of upland cotton plants treated with NaCl or KCl. **(A)** Phenotypes of upland cotton plants treated with no chloride (Mock), 200 mM NaCl, or 200 mM KCl for 10 days. **(B)** Cl^–^ contents (mg/g DW) of upland cotton plants (roots, stems, and leaves) treated with no chloride (Mock), 200 mM NaCl, or 200 mM KCl for 10 days. **(C)** Heatmap of the Cl^–^ contents of upland cotton plants treated with no chloride (Mock), 200 mM NaCl, or 200 mM KCl. The Cl^–^ concentrations are indicated by different colors and the colors are consistent in panels **(B,C)**.

Upland cotton plants were treated with 200 mM NaCl or KCl to assess the effects of chloride. After 10 days, compared with the mock controls, the growth of the plants treated with NaCl or KCl was significantly inhibited. Additionally, the salt-treated plants had shriveled and wilted leaves, some of which had fallen off the plants (Figure 1A).

An analysis of the plant ion contents indicated that the chloride (Cl^–^), sodium (Na^+^), and potassium (K^+^) contents were higher in the salt-treated samples than in the mock controls (Figures 1B,C and Supplementary Figure 1). After the NaCl and KCl treatments, cotton plants had extremely high Cl^–^ contents in the leaves, roots, and stems (Figures 1B,C). As expected, the Na^+^ or K^+^ contents also increased in the leaves, stems, and roots of plants treated with NaCl or KCl (Supplementary Figure 1). These results suggest that Cl^–^ can accumulate in cotton tissues, thereby affecting plant growth and development.

### Phylogenetic Analysis and Conserved Domains of *GhCLCg-1*

**FIGURE 2:**
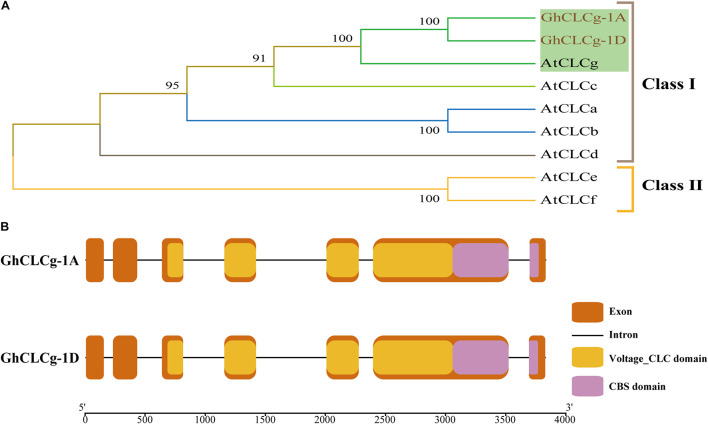
Phylogenetic analysis and gene structures of GhCLCg-1A/D. **(A)** Phylogenetic tree of GhCLCg-1A/D (*Gossypium hirsutum*) and AtCLCs (*Arabidopsis thaliana*). **(B)** Intron-exon and conserved domains structures of GhCLCg-1A/D. The exons, introns, Voltage_CLC domains and CBS domains are indicated by the orange boxes, black lines, yellow boxes, and purple boxes, respectively. The 5′-3′ scale indicates the size of DNA sequence. The exons/introns and domains sizes are proportional to DNA sequence lengths.

The vacuolar chloride channel (CLCg) can protect plant cells from ion-induced damages (Nguyen et al., 2015). Using a homology-based cloning strategy, we cloned the full-length coding sequences of two CLCg-encoding genes in *G. hirsutum*, which are homoeologs from D-subgenome and A-subgenome of the allotetraploid upland cotton and named *GhCLCg-1A/D* (suffixes D and A represented the D and A subgenomes, respectively). The *GhCLCg-1A* and *GhCLCg-1D* sequences were 2,325 bp long and encoded 774 amino acids. To further characterize *GhCLCg-1A/D*, we used the MEGA-X program to construct a phylogenetic tree that included seven AtCLCs. In the constructed tree, the CLC family was divided into two clades, with GhCLCg belonging to class I and highly homologous to AtCLCg (Figure 2A).

The results of the *GhCLCg-1A* and *GhCLCg-1D* gene structural analysis revealed these two genes have a similar intron-exon arrangement (Figure 2B). Specifically, they both comprise seven exons and six introns as well as domains unique to the CLC family (i.e., voltage_CLC and CBS). To further investigate the similarity in the *GhCLCg-1A* and *GhCLCg-1D* sequences, their amino acid sequences were aligned to the AtCLCg amino acid sequence (Supplementary Figure 2). The multiple sequence alignment confirmed that GhCLCg-1A and GhCLCg-1D are highly conserved (98.19% identity) and are similar to AtCLCg at the amino acid level (91.13% identity). The multiple sequence alignment also indicated that GhCLCg-1A and GhCLCg-1D have three regions that are conserved in AtCLCg, namely GxGIPE, GKxGPxxH, and PxxGxLF. In the GxGIPE sequence, x was a serine (S), implying GhCLCg-1A/D are specific for Cl^–^ (Supplementary Figure 2) (Barbier-Brygoo et al., 2000; Dutzler et al., 2002; Zifarelli and Pusch, 2010). These results imply that GhCLCg-1A and GhCLCg-1D are functionally similar to AtCLCg.

### Subcellular Localization of *GhCLCg-1*

**FIGURE 3:**
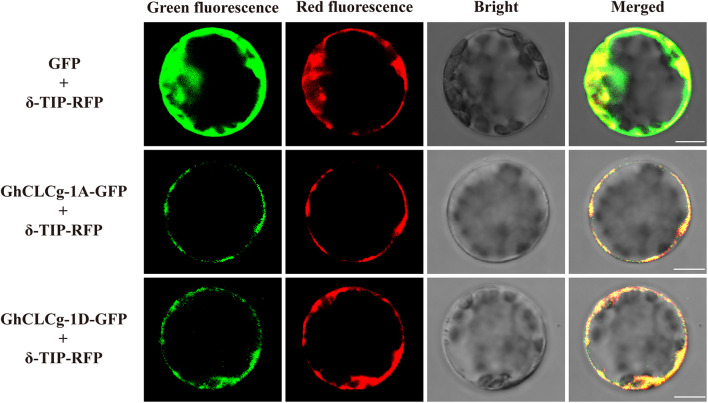
Subcellular localization of GhCLCg-1A/D. GhCLCg-1A-GFP, and GhCLCg-1D-GFP were co-expressed with the vacuolar marker protein δ-TIP-red fluorescent protein (RFP) in leaf protoplasts of *Arabidopsis thaliana* seedlings. Green fluorescent protein (GFP) co-transformed with δ-TIP-RFP as positive control. Scale bar = 10 μM.

The localization of proteins provides clues regarding their functions (Long et al., 2020). The subcellular localization of GhCLCg-1A/D was determined using fusion proteins with a C-terminal GFP. The fusion proteins were transiently expressed in *A. thaliana* leaf protoplasts that also contained the vacuolar marker protein δ-TIP with a C-terminal RFP (Figure 3). In the control protoplasts with GFP alone and δ-TIP-RFP, the GFP signal was detected throughout, whereas the RFP fluorescence was restricted to the tonoplast. The GhCLCg-1A/D-GFP fusion proteins co-localized with δ-TIP-RFP in the protoplast, with red and green fluorescent signals overlapping, which confirmed that GhCLCg-1A/D are localized to the vacuolar membrane.

### Activation of *GhCLCg-1* by Chloride in Upland Cotton Plants

**FIGURE 4:**
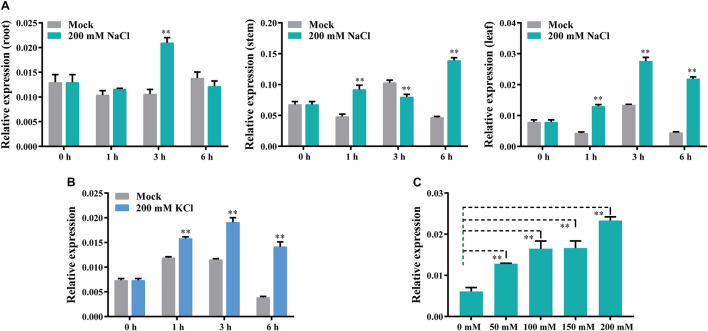
*GhCLCg-1* expression profiles in salt-treated upland cotton plants. *GhCLCg-1* expression patterns in the roots, stems, and leaves of plants treated with 200 mM NaCl for 0, 1, 3, and 6 h **(A)** or in the leaves of plants treated with 200 mM KCl for 0, 1, 3, and 6 h **(B)**. **(C)** *GhCLCg-1* expression patterns in the leaves of plants treated for 6 h with different NaCl concentrations (0, 50, 100, 150, and 200 mM). The 2^–ΔCT^ method was used to calculate relative expression levels. Error bars indicate the standard deviation (SD) of three biological replicates (^∗∗^*p* < 0.01; *t*-test).

To determine whether *GhCLCg-1A/D* are involved in salt stress responses, we analyzed the expression of the corresponding genes following an exposure to chloride salts. The substantial similarity in the *GhCLCg-1A* and *GhCLCg-1D* sequences makes it very difficult to study the expression of these two genes by qRT-PCR. In this study, the universal qRT-PCR primers GhCLCg-1-Q-F/R were designed to analyze *GhCLCg-1A/D* expression in the roots, stems, and leaves at various time-points (Figure 4). Following the 200 mM NaCl treatment, *GhCLCg-1* expression was up-regulated at 3 h in the roots (approximately two times higher than expression level of the mock control). In the stems, *GhCLCg-1* expression was about 2–3 times higher in the NaCl-treated plants than in the mock controls at 1 and 6 h. Unexpectedly, the expression level of *GhCLCg-1* at 3 h in stems of the 200 mM NaCl treated plants was significantly lower than in stems of the mock plants. The *GhCLCg-1* transcript levels in stems treated with different salt concentration at 3 h were analyzed. The results showed that the expression of *GhCLCg-1* at different salt concentration is lower than the control (0 mM NaCl), but the expression is increased by high salt concentration, and the expression level under 200 mM NaCl is higher than under 50, 100, and 150 mM NaCl (Supplementary Figure 3). In the leaves, *GhCLCg-1* expression was up-regulated by the salt treatment at 1, 3, and 6 h. More specifically, the *GhCLCg-1* transcript level was highest at 3 h, but the increase (relative to expression level of the mock control) was greatest at 6 h (five times higher) (Figure 4A). To further demonstrate that *GhCLCg-1* is involved in plant responses to chloride-mediated salt stress, cotton plants were treated with 200 mM KCl (Figure 4B). The changes in the leaf *GhCLCg-1* expression levels under 200 mM KCl were similar to those resulting from the 200 mM NaCl treatment. Given the up-regulated *GhCLCg-1* expression induced by 200 mM NaCl, the leaf *GhCLCg-1* expression levels following 6 h treatments with various NaCl concentrations (0, 50, 100, 150, and 200 mM) were analyzed. The relative *GhCLCg-1* expression level increased as the salt concentration increased, peaking at 200 mM NaCl (Figure 4C). Thus, *GhCLCg-1* expression in leaves was induced and regulated by chloride-mediated salt stress. Moreover, this expression was positively related to the chloride concentration.

### Overexpression of *GhCLCg-1* in *Arabidopsis thaliana* Enhanced Salt Tolerance

**FIGURE 5:**
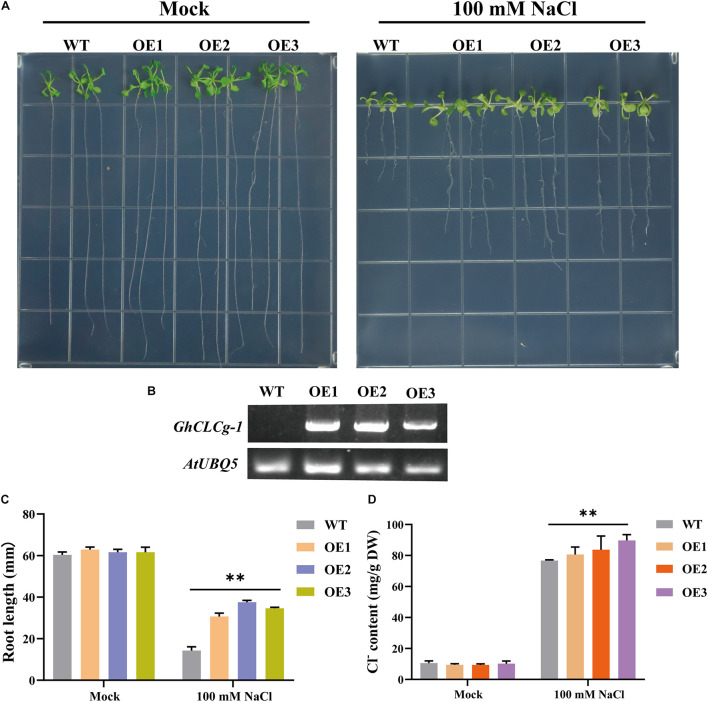
Effects of overexpressing of *GhCLCg-1* in *Arabidopsis thaliana*. **(A)** Wild-type (WT) and *GhCLCg-1*-overexpressing transgenic *A. thaliana* lines (OE1, OE2, and OE3) grown on Murashige and Skoog (MS) medium (Mock) and MS medium containing 100 mM NaCl for 21 days. **(B)** Reverse transcription PCR analysis of *GhCLCg-1* expression levels in WT, OE1, OE2, and OE3 plants, with *AtUBQ5* used as an internal control. **(C)** Comparison of the WT, OE1, OE2, and OE3 root lengths. **(D)** Comparison of the WT, OE1, OE2, and OE3 chloride contents. Data are presented as the mean ± standard deviation (SD) of three biological replicates (^∗∗^*p* < 0.01, *t*-test).

To further clarify the *GhCLCg-1* function related to chloride tolerance, *GhCLCg-1*-overexpressing (OE) transgenic *A. thaliana* plants were generated and examined (Figure 5). The WT, OE1, OE2, and OE3 *A. thaliana* plants had similar growth phenotypes on MS medium (Mock), with no significant differences in the root length and shoot growth. However, when treated with 100 mM NaCl, the transgenic *A. thaliana* plants (OE1, OE2, and OE3) had larger leaves and longer roots than the WT plants (Figure 5A). A quantitative analysis of the root length under salt stress conditions confirmed that the transgenic *A. thaliana* plants had significantly longer roots than the WT plants (Figure 5C). The *GhCLCg-1* expression level increased significantly in the transgenic *A. thaliana* plants, whereas *GhCLCg-1* expression was undetectable in the WT controls (Figure 5B). The changes in the Cl^–^ contents in *A. thaliana* plants (WT, OE1, OE2, and OE3) were analyzed. Under normal conditions, there were no significant differences in the Cl^–^ contents between the WT and transgenic plants. However, the Cl^–^ contents were significantly higher in the transgenic plants than in the WT controls under salt stress conditions (Figure 5D). These results indicate that *GhCLCg-1* confers salt tolerance, while also altering the uptake of Cl^–^ in plants.

**FIGURE 6:**
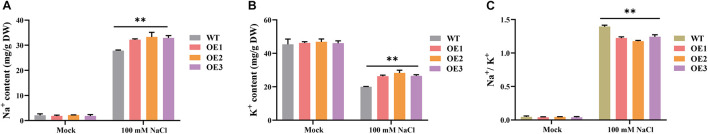
Cation contents of wild-type (WT) and *GhCLCg-1*-overexpressing transgenic *Arabidopsis thaliana* lines (OE1, OE2, and OE3). **(A)** Na^+^ contents, **(B)** K^+^ contents, and **(C)** Na^+^/K^+^ ratio of *A. thaliana* under 0 mM or 100 mM NaCl treatment for 21 days. Data are presented as the mean ± standard deviation (SD) of three biological replicates (^∗∗^*p* < 0.01, *t*-test).

High environmental Na^+^ and Cl^–^ levels alter the ion homeostasis of plant cells, resulting in an ionic imbalance and toxicity. Thus, re-establishing cellular ion homeostasis is critical for metabolic functions and growth (Niu et al., 1995). The changes in Na^+^ and K^+^ contents as well as the Na^+^/K^+^ ratio in WT and transgenic *A. thaliana* plants (OE1, OE2, and OE3) were analyzed (Figure 6). There were no significant differences in the Na^+^ and K^+^ contents or the Na^+^/K^+^ ratio among the WT, OE1, OE2, and OE3 plants on MS medium (Mock). An examination of the effects of the 100 mM NaCl treatment revealed that compared with the WT controls, the Na^+^ and K^+^ contents were significantly higher in the OE1, OE2, and OE3 plants, whereas the Na^+^/K^+^ ratios were significantly lower in the OE1, OE2, and OE3 plants. These findings suggest that overexpression of *GhCLCg-1* can affect the accumulation of Na^+^ and K^+^, then leads to a decrease of the Na^+^/K^+^ ratios under the NaCl treatment, which is beneficial for plant tolerance to salt.

### Silencing of *GhCLCg-1* in Upland Cotton Compromised Salt Tolerance

**FIGURE 7:**
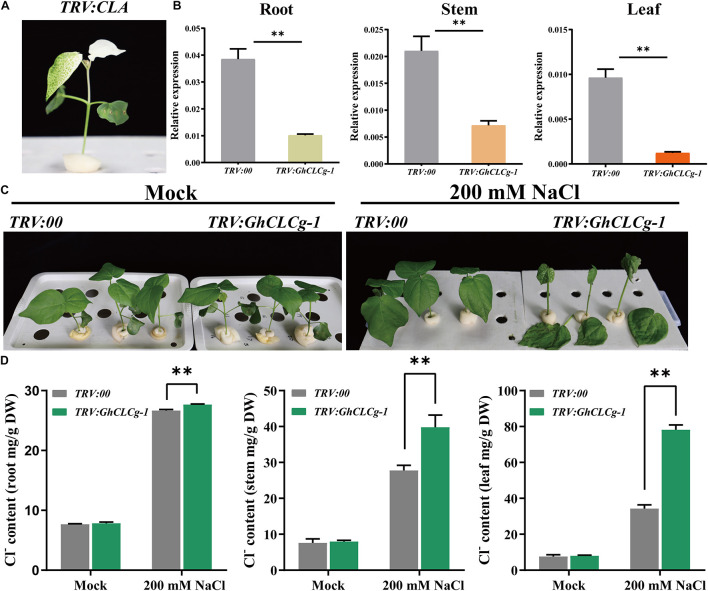
Effects of silencing *GhCLCg-1* in upland cotton. **(A)** The *TRV:CLA* plants (positive control) exhibited an albino phenotype. **(B)** Efficiency of the silencing of *GhCLCg-1* in *TRV:00* and *TRV:GhCLCg-1* plants analyzed by qRT-PCR with *GhHIS3* used as an internal control. **(C)** Phenotypes of *TRV:00* and *TRV:GhCLCg-1* plants treated with 0 mM (Mock) or 200 mM NaCl for 3 days. **(D)** Cl^–^ contents of *TRV:00* and *TRV:GhCLCg-1* plants after the salt treatment. Data are presented as the mean ± standard deviation (SD) of three biological replicates (^∗∗^*p* < 0.01, *t*-test).

To more thoroughly investigate the roles of *GhCLCg-1* related to the salt tolerance of upland cotton plants, we used the TRV-based VIGS system to generate *GhCLCg-1* knockdown cotton plants (Figure 7). At 10 days after the *A. tumefaciens* infiltration, the cotton seedlings transformed with *TRV:CLA* exhibited an albino phenotype (Figure 7A). The *GhCLCg-1* expression levels were determined by qRT-PCR to assess how efficiently the gene was silenced in the cotton plants. The *GhCLCg-1* expression levels in the roots, stems, and leaves were obviously lower in the *TRV:GhCLCg-1* plants than in the *TRV:00* plants (Figure 7B). Accordingly, *GhCLCg-1* was efficiently silenced in the *TRV:GhCLCg-1* cotton plants.

There were no observable differences in the growth of *TRV:00* and *TRV:GhCLCg-1* plants under normal conditions (Mock). However, after a 3-day 200 mM NaCl treatment, several symptoms consistent with salt stress were detected on the *TRV:GhCLCg-1* plants (Figure 7C). Specifically, the leaves were shrunken and wilted and some of the leaves even fell from the plants. A decrease in the *GhCLCg-1* expression level increased the sensitivity of plants to salt stress, suggesting *GhCLCg-1* influences cotton salt tolerance. Furthermore, we analyzed the Cl^–^ contents in the roots, stems, and leaves of the *TRV:00* and *TRV:GhCLCg-1* cotton plants (Figure 7D). As expected, in the absence of chloride stress, there were no significant differences in the Cl^–^ contents of *TRV:GhCLCg-1* and *TRV:00* plants. However, following the 200 mM NaCl treatment, the root, stem, and leaf Cl^–^ contents were significantly higher in the *TRV:GhCLCg-1* plants than in the *TRV:00* plants. A comparison of these three tissues in *TRV:GhCLCg-1* plants revealed that the Cl^–^ contents were highest in the leaves, followed by the stems and then the roots. Compared with the *TRV:00* plants, the *GhCLCg-1*-silenced plants accumulated more Cl^–^, especially in the leaves, making them more sensitive to salt stress.

**FIGURE 8:**
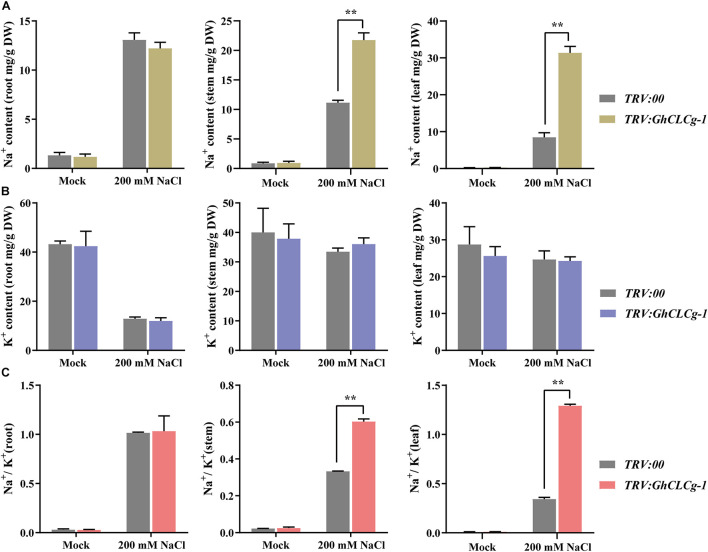
Cation contents of *TRV:00* and *TRV:GhCLCg-1* plants. **(A)** Na^+^ contents, **(B)** K^+^ contents, and **(C)** Na^+^/K^+^ ratio of *TRV:00* and *TRV:GhCLCg-1* plants with salt treated for 3 days. Data are presented as the mean ± standard deviation (SD) of three biological replicates (^∗∗^*p* < 0.01, *t*-test).

The Na^+^ and K^+^ contents as well as the Na^+^/K^+^ ratio in *TRV:00* and *TRV:GhCLCg-1* plants were measured. There were no significant differences in the Na^+^ and K^+^ contents or the Na^+^/K^+^ ratio between the *TRV:00* and *TRV:GhCLCg-1* plants in the absence of salt stress (Figure 8). In contrast, after the 200 mM NaCl treatment, the shoot (stems and leaves) Na^+^ content was higher in the *TRV:GhCLCg-1* plants than in the *TRV:00* plants, and the Na^+^ content was highest in the *TRV:GhCLCg-1* leaves (Figure 8A). The NaCl treatment did not significantly affect the K^+^ contents of the *TRV:00* and *TRV:GhCLCg-1* shoots, but it obviously decreased the K^+^ contents of the *TRV:00* and *TRV:GhCLCg-1* roots (Figure 8B). Interestingly, the Na^+^/K^+^ ratios sharply and significantly increased in the stems and leaves of *TRV:GhCLCg-1* plants in response to the salt treatment, and the Na^+^/K^+^ ratio was highest in the *TRV:GhCLCg-1* leaves (Figure 8C). These results indicate that silencing *GhCLCg-1* causes cotton plants to absorb Na^+^ and accumulate in shoots, then leads to an increased Na^+^/K^+^ ratio in stems and leaves under NaCl treatment, which increases sensitivity to salinity stress.

## Discussion

Many studies showed that salt ions can induce osmotic stress and ion toxicity in plants and restrict their growth (Munns and Tester, 2008). Moreover, soil salinity interferes with water and nutrient uptake by the roots, and salt ions accumulate more in leaves than in roots (Tester and Davenport, 2003). In the current study, we proved that chloride salt stress causes cotton plants to accumulate more salt ions (Cl^–^, Na^+^, and K^+^), especially in the leaves. Cotton plant growth and development were significantly inhibited by high salinity (Figure 1 and Supplementary Figure 1).

Previous studies revealed that AtCLCg in *A. thaliana* is localized to the tonoplast and modulates chloride homeostasis in response to chloride stress, with shoot chloride levels increasing in *atclcg* knockout mutants (Nguyen et al., 2015). In the current study, we cloned a chloride channel gene (GhCLCg-1A/D) in upland cotton. Analysis of amino acid sequences revealed GhCLCg-1A/D belong to the CLCg subfamily and contain the conserved amino acid sequence motif (GSGIPE) associated with chloride selectivity (Atsuko et al., 2006; Li et al., 2006; Zifarelli and Pusch, 2010). The qRT-PCR data verified that *GhCLCg-1* is responsive to chloride salt stress in leaves. At the transcriptional level, *GhCLCg-1* was similarly affected by 200 mM KCl and 200 mM NaCl (Figures 4A,B). The leaf *GhCLCg-1* transcription level increased as the treatment duration and chloride concentration increased (Figures 4A,C). These results imply *GhCLCg-1* is involved in the chloride-mediated salt stress response in upland cotton plants.

Plants can minimize the effects of salt stress by eliminating excess salt ions through plasma membranes or by sequestering them in vacuoles (Zelm et al., 2020). The biological roles of plant proteins are closely related to their subcellular localization (Long et al., 2020). Our study confirmed that GhCLCg-1A/D are localized to the tonoplast (Figure 3), suggesting they may contribute to the transport of Cl^–^ into vacuoles to participate in plant response to chloride salt. In a previous study, *GmCLC1*-overexpressing soybean plants sequestered more Cl^–^ in the roots and transported less Cl^–^ to the shoots than the WT controls, which enhanced the salt tolerance of the transgenic plants (Wei et al., 2016). The overexpression of the vacuolar chloride channel gene *GsCLC-c2* influences Cl^–^ homeostasis. More specifically, it improves salt tolerance by increasing the accumulation of Cl^–^ in the roots, thereby decreasing the amount of Cl^–^ transported to the shoots. Using two-electrode voltage clamps and *Xenopus laevis* oocytes, a previous study proved that *GsCLC-c2* mediates the transport of Cl^–^ (Wei et al., 2019). The insertion of the vacuolar chloride channel gene *OsCLC-1* into *gef1* yeast cells, in which the single putative chloride channel is mutated, decreases the relative sensitivity of yeast to various chloride salts (Atsuko et al., 2006).

In this study, our qRT-PCR analysis suggested that tonoplast-localized *GhCLCg-1* is involved in chloride stress responses (Figure 4), which is consistent with the findings of a recent investigation (Liu et al., 2020). The overexpression of *GhCLCg-1* in *A. thaliana* can enhance salt tolerance and alter Cl^–^ accumulation (Figure 5). During an exposure to NaCl stress, most of the Cl^–^ is not in vacuoles, but is in the cytoplasm or the apoplast, where it inhibits enzyme activities or increases water loss, respectively, in the absence of AtCLCg (Flowers et al., 2010; Teakle and Tyerman, 2010). The overexpression of the chloride channel gene *CsCLCc* in *A. thaliana* increases the salt tolerance of the transgenic plants. Moreover, the root and shoot Cl^–^ contents are lower in the transgenic plants than in the mutant or WT plants (Wei et al., 2013). In this study, the transgenic *A. thaliana* plants overexpressing *GhCLCg-1* accumulated more Cl^–^ than the WT controls (Figure 5C). This is in contrast to the Cl^–^ accumulation in the *CsCLCc*-overexpressing *A. thaliana* plants examined in an earlier investigation (Wei et al., 2013), but it is similar to the reported changes to the Cl^–^ contents in *A. thaliana atclcg* mutants (Nguyen et al., 2015). It could be deduced that the results might be related to the role of CLCg. In *A. thaliana atclcg* mutants, loss of function of *AtCLCg* would result in accumulating more Cl^–^ in the cytoplasm, because Cl^–^ transport to the vacuole was disrupted, which increased Cl^–^ contents and compromised salt tolerance. However, in the *A. thaliana* plants overexpressing *GhCLCg-1*, the extra Cl^–^ was transported and accumulated in the vacuole by *GhCLCg-1* to reduce its toxic effects in the cytoplasm, which also increased Cl^–^ contents, but enhanced salt tolerance. Similar to the *A. thaliana atclcg* mutants, silencing *GhCLCg-1* expression *via* VIGS also increased the Cl^–^ contents and the sensitivity of cotton plants to chloride salt (Figure 7). Notably, *TRV:GhCLCg-1* plants accumulated more Cl^–^ in the leaves than in the roots under salt stress conditions, which was consistent with previous studies that *A. thaliana clc* mutants have more Cl^–^ in the shoots than in other tissues (Jossier et al., 2010; Nguyen et al., 2015). The ion homeostasis is crucial for the salt tolerance of higher plants (Li et al., 2006). Furthermore, a low Na^+^/K^+^ ratio may maintain cell metabolic activities as well as salt tolerance (Blumwald, 2000). Although more Na^+^ and K^+^ were accumulated in the *A. thaliana* plants overexpressing *GhCLCg-1*, the Na^+^/K^+^ ratio was still lower than that of the WT plants under 100 mM NaCl (Figure 6). By contrary, the stem and leaf accumulated more Na^+^ in the *TRV:GhCLCg-1* plants, leading to a higher Na^+^/K^+^ ratios than that in the *TRV:00* plants after treatment with 200 mM NaCl (Figure 8). Considered together, these observations reveal that the *GhCLCg-1* play a vital role in salt tolerance of upland cotton by regulating ion accumulation.

## Conclusion

In conclusion, we revealed upland cotton plants accumulated Cl^–^ and were damaged by chloride salt stress. Gene expression profiles suggested that the expression of *GhCLCg-1*, which encodes a tonoplast-localized chloride channel, is up-regulated in response to chloride salt stress. The overexpression of *GhCLCg-1* in *A. thaliana* altered the accumulation of ions with a decrease of the Na^+^/K^+^ ratios, and enhanced salt tolerance. Moreover, cotton plants, in which *GhCLCg-1* was silenced, accumulated more Cl^–^ and increased the Na^+^/K^+^ ratios, leading to greater sensitivity to chloride salt. These results indicate that upland cotton plants accumulate Cl^–^ under salt conditions and *GhCLCg-1* alleviates damages resulting from excessive amounts of chloride salt.

## Data Availability Statement

The datasets presented in this study can be found in online repositories. The names of the repository/repositories and accession number(s) can be found in the article/Supplementary Material.

## Author Contributions

WL and ZM conceived and designed the research. WL, JF, and WM performed the experiments and analyzed the data. ZM provided the materials. WL and JF prepared the figures and wrote the manuscript. ZM and YZ revised the manuscript. All authors have read and approved the final manuscript.

## Conflict of Interest

The authors declare that the research was conducted in the absence of any commercial or financial relationships that could be construed as a potential conflict of interest.

## Publisher’s Note

All claims expressed in this article are solely those of the authors and do not necessarily represent those of their affiliated organizations, or those of the publisher, the editors and the reviewers. Any product that may be evaluated in this article, or claim that may be made by its manufacturer, is not guaranteed or endorsed by the publisher.

## Footnotes

1
https://www.cottongen.org/

2
https://www.arabidopsis.org/

3
http://www.ebi.ac.uk/interpro/

## Supplementary Material

Supplementary Figure 1 Cation contents in upland cotton plants. Na^+^ contents (mg/g DW) of upland cotton plants (roots, stems, and leaves) treated with no chloride (Mock) **(A)** or 200 mM NaCl **(B)** for 10 days. K^+^ contents (mg/g DW) of upland cotton plants (roots, stems, and leaves) treated with no chloride (Mock) **(C)** or 200 mM KCl **(D)** for 10 days. **(E)** Heatmap of the cation contents of upland cotton plants treated with no chloride (Mock), 200 mM NaCl, or 200 mM KCl. The cation concentrations are indicated by different colors and the colors are consistent in panels **(A–E)**.

Supplementary Figure 2 Sequence alignment of the GhCLCg-1A, GhCLCg-1D, and AtCLCg. Conserved residues GxxGIPE, GKxGPxxH, and PxxGxLF are indicated by the red, blue, and green boxes, respectively.

Supplementary Figure 3 *GhCLCg-1* expression patterns in the stems of plants treated for 3 h with different NaCl concentrations (0, 50, 100, 150, and 200 mM). The 2^–ΔCT^ method was used to calculate relative expression levels. Error bars indicate the standard deviation (SD) of three biological replicates (^∗∗^*p* < 0.01; *t*-test).

Supplementary Table 1 Primers used in this study.

Supplementary Table 2 Genes used in this study.
