# Supplementary figures and images for: GhCLCg-1, a Vacuolar Chloride Channel, Contributes to Salt Tolerance by Regulating Ion Accumulation in Upland Cotton
Source: Front Plant Sci. 2021 Oct 15;12:765173. doi: 10.3389/fpls.2021.765173 (PMC8555695; doi:10.3389/fpls.2021.765173)

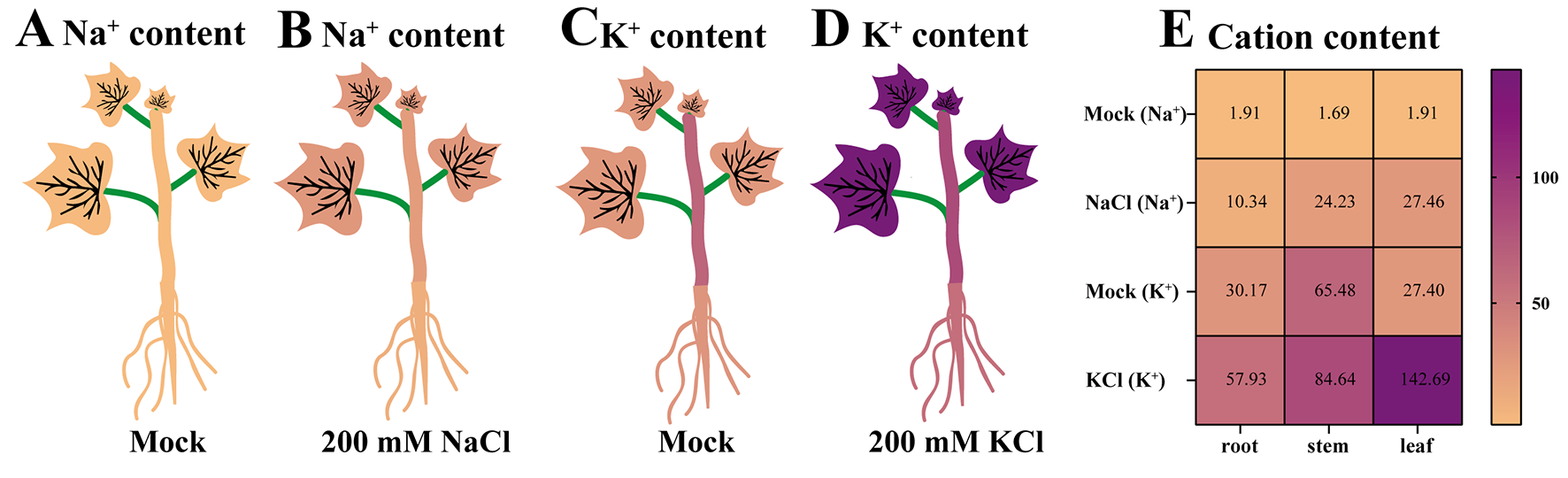

Supplement: Supplementary Figure 1 — Cation contents in upland cotton plants. Na+ contents (mg/g DW) of upland cotton plants (roots, stems, and leaves) treated with no chloride (Mock) (A) or 200 mM NaCl (B) for 10 days. K+ contents (mg/g DW) of upland cotton plants (roots, stems, and leaves) treated with no chloride (Mock) (C) or 200 mM KCl (D) for 10 days. (E) Heatmap of the cation contents of upland cotton plants treated with no chloride (Mock), 200 mM NaCl, or 200 mM KCl. The cation concentrations are indicated by different colors and the colors are consistent in panels (A–E). [file Image_1.TIF]

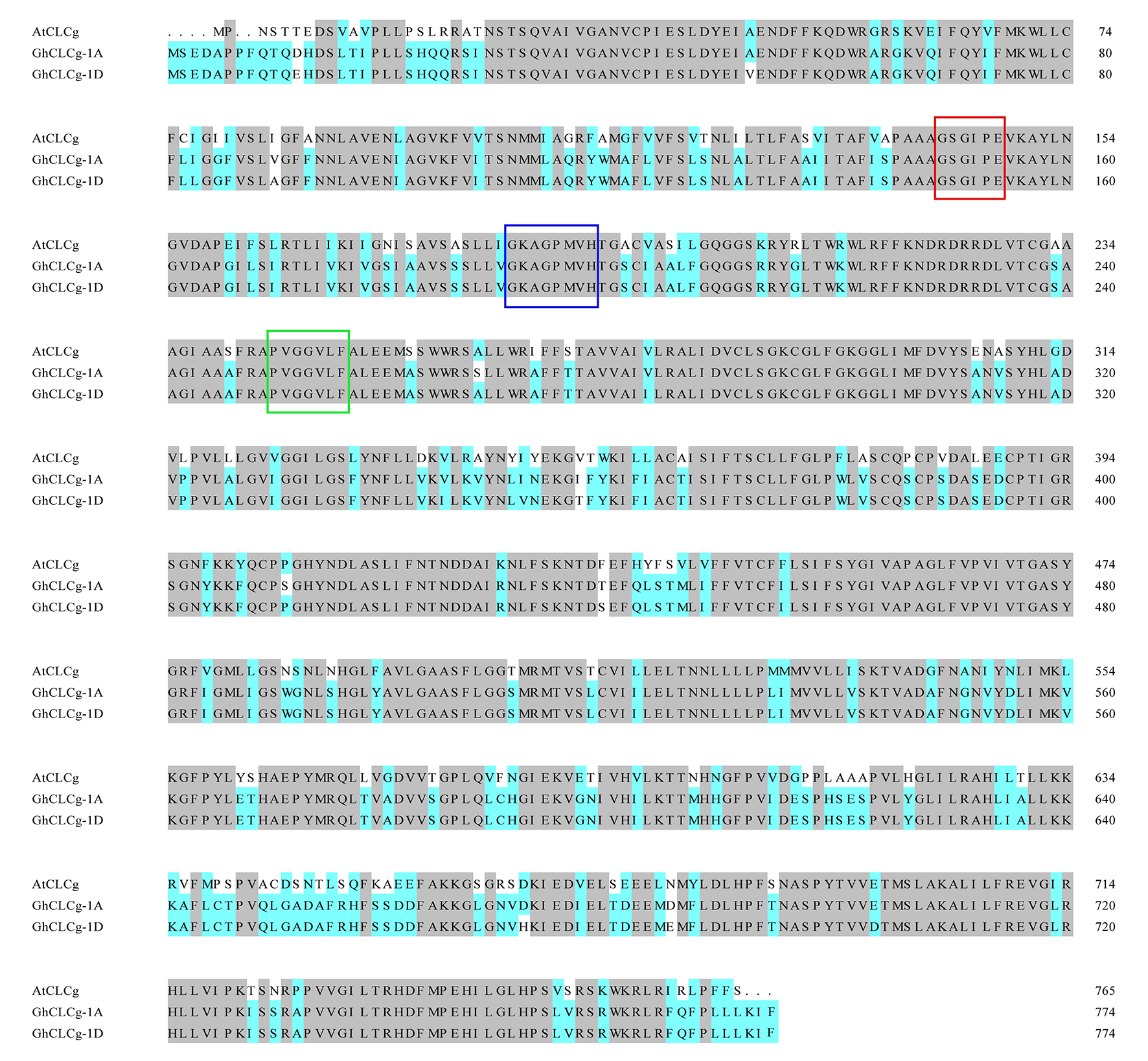

Supplement: Supplementary Figure 2 — Sequence alignment of the GhCLCg-1A, GhCLCg-1D, and AtCLCg. Conserved residues GxxGIPE, GKxGPxxH, and PxxGxLF are indicated by the red, blue, and green boxes, respectively. [file Image_2.tif]

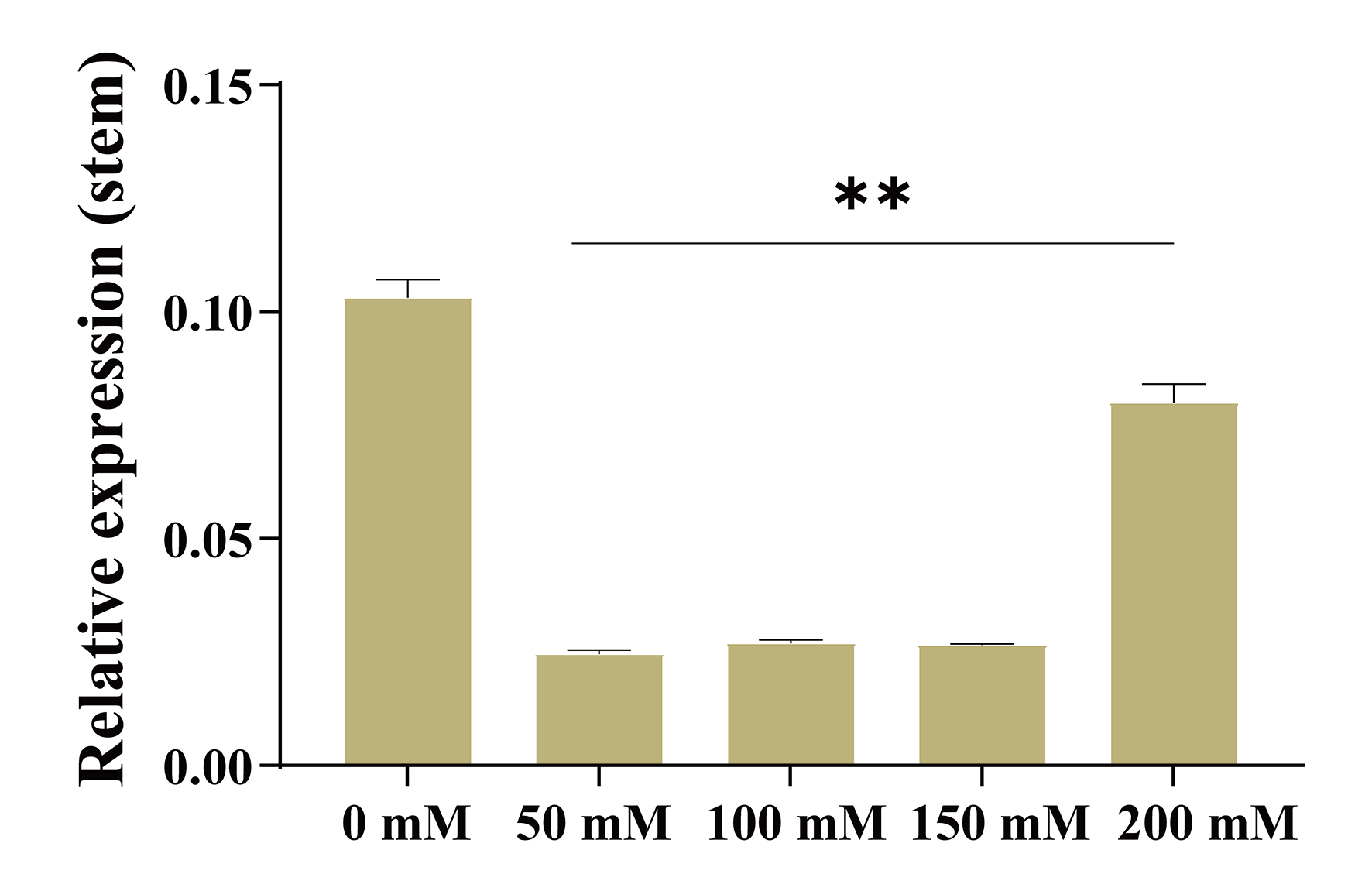

Supplement: Supplementary Figure 3 — GhCLCg-1 expression patterns in the stems of plants treated for 3 h with different NaCl concentrations (0, 50, 100, 150, and 200 mM). The 2–ΔCT method was used to calculate relative expression levels. Error bars indicate the standard deviation (SD) of three biological replicates (∗∗p < 0.01; t-test). [file Image_3.TIF]
